# Tobacco and electronic cigarette smoking among in-school adolescents in Vietnam between 2013 and 2019: prevalence and associated factors

**DOI:** 10.1080/16549716.2022.2114616

**Published:** 2022-09-29

**Authors:** Hoang Van Minh, Khuong Quynh Long, Do Van Vuong, Nguyen Manh Hung, Kidong Park, Momoe Takeuchi, Mina Kashiwabara, Nguyen Tuan Lam, Pham Thi Quynh Nga, Le Phuong Anh, Le Van Tuan, Tran Quoc Bao, Le Duong Minh Anh, Tran Thi Tuyet Hanh

**Affiliations:** a Hanoi University of Public Health, Hanoi, Vietnam; b Data, Strategy and Innovation, World Health Organization, Regional Office for the Western Pacific, Manila, Philippines; c Country Liaison Officer, World Health Organization Office for Northern Micronesia, Federated States of Micronesia, Marshall Islands and Palau; d Non-Communicable Diseases, World Health Organization Division of Pacific Technical Support; e Universal Health Coverage – Healthy Lifestyle and Environment, World Health Organization, Country Office for Viet Nam, Hanoi, Vietnam; f Department of Epidemiology, Harvard T.H. School of Public Health, Harvard University, Massachusetts, United States of America; g Physical Education Department, Ministry of Education and Training, Hanoi, Vietnam; h General Department of Preventive Medicine, Ministry of Health, Hanoi, Vietnam

**Keywords:** Adolescent health, risk behaviour, nicotine vaping, tobacco use, GSHS

## Abstract

**Background:**

Smoking among adolescents in schools is a major global public health concern. There is limited evidence regarding prevalence and associated factors in Vietnam.

**Objective:**

To compare the prevalence of smoking and associated factors among in-school adolescents aged 13–17 years in Vietnam between 2013 and 2019.

**Methods:**

Data were collected from two rounds of the national representative Vietnam Global School-based Student Health Survey (GSHS) conducted in 2013 (n = 3,331) and 2019 (n = 7,690). Logistic regression was used to identify the factors associated with tobacco and electronic cigarette smoking among in-school adolescents.

**Results:**

There was a significant reduction in the prevalence of current smoking (water pipes and cigarettes) from 5.4% (95% CI: 4.0–7.2) in 2013 to 2.8% (95% CI: 2.2–3.6) in 2019. In 2019, 2.6% of the in-school adolescents reported having used electronic cigarette products 30 days prior to the survey. Factors associated with a significantly higher likelihood of current smoking status included gender, loneliness, suicidal ideation, sexual activity, truancy, and alcohol consumption. Similar patterns were observed for e-cigarettes.

**Conclusion:**

Smoking among in-school adolescents in Vietnam decreased between 2013 and 2019. Follow-up studies are needed to further investigate causal factors so that future policies and communication programmes can be more effectively targeted to reduce smoking in adolescents.

## Background

Tobacco smoking is a dangerous risk behaviour, potentially leading to many adverse human health consequences, including cancers, cardiovascular and chronic pulmonary diseases, and many other adverse health problems [1]. Globally, tobacco smoking is responsible for more than eight million deaths annually, and this is expected to increase [1]. Approximately 80% of the world’s 1.3 billion smokers live in low- and middle-income countries (LMICs), where the tobacco-related burden of the disease is greater than in high-income countries [1].

Global evidence shows that the average age of first-time tobacco use is declining [2–6]. A study in Timor-Leste found that more than 50% of current smokers started smoking in adolescence [3]. A study among school adolescents in India showed that the mean age of smoking initiation was approximately twelve and a half. In an Indian study, nearly 70% of boys and 80% of girls aged younger than 15 years reported beginning smoking tobacco before the age of eleven [2]. The proportion of students taking up smoking in early adulthood in the USA (US) doubled between 2002 (20.6%) and 2018 (42.6%) [4]. According to the Global Youth Tobacco Survey, which was conducted in 61 countries between 2012 and 2015, the median proportion of current smokers among students aged 13–15 was 10.7% [7,8]. Evidence also shows that adolescents who begin smoking before the age of 18 are more likely to find it harder to quit than those who begin later in life [9].

Vietnam is one of the 15 countries with the highest proportion of male smokers. The prevalence of smoking among adults aged ≥ 15 years in Vietnam in 2015 was 22.5% (45.3% among men and 1.1% among women) [10]. Approximately 75,000 people in Vietnam die each year from complications caused by smoking [11]. Although the prevalence of smoking among adolescents in Vietnam is relatively low at 3.5% (6.3% among boys and 0.9% among children aged 13–15) [12], the issue of youth smoking in Vietnam should not be underestimated. Tobacco companies intentionally market and promote cigarette smoking among adolescents [9]. Moreover, information on smoking rates among Vietnamese adolescents is limited because most previous surveys have focused on adult smoking habits.

Due to this lack of evidence, there has been little policy interest in adolescent smoking in Vietnam. This study used data from two rounds of the Vietnam Global School-based Student Health Survey (GSHS) conducted in 2013 and 2019. The objective was to estimate the prevalence of smoking, including tobacco and electronic cigarettes (e-cigarettes), among in-school adolescents in Vietnam. The study also assessed factors associated with the smoking status of in-school adolescents to identify the high-risk groups and potential protective factors to inform future prevention programmes.

## Methods

### Data source

The Vietnam GSHS conducted in 2013 and 2019 used the standardised protocol of the World Health Organization (WHO) which was designed to collect data from students in developing countries [13]. Technical support was provided by the Centre for Disease Control and Prevention in the US. The Vietnamese GSHS employed a two-stage cluster sampling design to produce a nationally representative sample of in-school adolescents aged 13–17. First, schools were selected based on a probability proportional to the enrolment size. Second, in each school, classes were chosen using a simple random sampling technique. All students in each selected class were invited to participate in the survey. The final sample size for GSHS 2013 was 3,331 in-school adolescents from 13 cities/provinces (response rate 96.0%), while the sample size for GSHS 2019 was 7,796 in-school adolescents from 20 cities/provinces (response rate 93.5%).

### Questionnaire development

The GSHS standardised questionnaire [13] was translated from English to Vietnamese using forward and backward translations for the Vietnam GSHS 2013 survey. The 2013 translated questionnaire was pilot tested with 50 in-school adolescents before use. For GSHS 2019, the 2013 version was updated as per the 2019 original English version. Relevant indicators from the 25 Global Non‐Communicable Diseases and Healthy Vietnam documents [14] were adapted for use. The 2019 questionnaire was pilot tested with 120 in-school adolescents aged 13–17. The pilot data were reviewed by senior researchers for the comprehensibility of questions, appropriateness of language and structural logic. Suggested modifications were undertaken to improve clarity.

### Data collection

In each selected class, a homeroom teacher explained the study to students. Consent forms for the students were collected with the consent of their parents or guardians. The following day, the GSHS questionnaires were administered to students who provided parental consent and were willing to participate in the study. Data collectors (medical or public health students trained on both the GSHS questionnaire and the data collection procedure) were responsible for the data collection process. In-school adolescents answered a self-administered questionnaire on separate computer-scannable answer sheets during a single classroom period. All data collection procedures adhered to WHO guidelines and templates.

### Variables

The dependent variable was self-reported current smoking status (defined as reporting having smoked in the prior 30 days). The independent variables were demographic characteristics, including gender and age; parental factors, including parental monitoring, parental understanding, and parental respect; having close friends; mental health, including loneliness and suicidal ideation; violence; self-reported experiences of bullying in school; sexual intercourse; truancy; and risk behaviours, including alcohol consumption, sedentary lifestyle, and low fruit/vegetable intake. Participants’ smoking environment was also assessed. The questions and definitions of the study variables are described in Table 1.

**Table 1.** Definitions of dependent and independent variables analysed.

Variables	Question	Definition used in this paper
**Dependent variables**		
Smoked traditional tobacco	- During the past 30 days, on how many days did you smoke cigarettes? - During the past 30 days, on how many days did you use ‘thuoc lao’ (Vietnamese water pipe)? *1 ‘0 days’ to 7 ‘All 30 days’*	Students who smoked at least 1 time (cigarettes or Vietnamese water pipe) in the past 30 days *(Yes vs. No)*
Smoked e-cigarettes	During past 30 days, on how many days did you use electronic cigarettes? *1 ‘0 days’ to 7 ‘All 30 days’*	Students who smoked e-cigarettes at least 1 time in the past 30 days *(Yes vs. No)*
**Independent variables**		
Gender		Male vs. Female
Age	How old are you? 1 *‘13 year-old’ to* 5 *‘17 year-old or older’*	From 13 to 17
Parental monitoring	- During the past 30 days, how often did your parents or guardians check to see if your homework was done? - During the past 30 days, how often did your parents or guardians really know what you were doing with your free time? 1 *‘Never’*; 2 *‘Rarely’*; 3 *‘Sometimes’*; 4 *‘Most of the time*’; 5 *‘Always’*	Students whose parents most of the time/always checked homework or know what their children do in their free time *(High vs. Low)*
Parental understanding	- During the past 30 days, how often did your parents or guardians understand your problems and worries? - During the past 30 days, how often did your parents or guardians give you advice and guidance? 1 *‘Never’*; 2 *‘Rarely’*; 3 *‘Sometimes’*; 4 *‘Most of the time’;* 5 *‘Always’*	Students whose parents most of the time/always understood problems/worries or gave advice/guidance *(High vs. Low)*
Parental respect	- During the past 30 days, how often did your parents or guardians go through your things without your approval? - During the past 30 days, how often did your parents or guardians not respect you as a person (for example, not let you talk or favour someone else more than you)? 1 *‘Never’*; 2 *‘Rarely’*; 3 *‘Sometimes’*; 4 *‘Most of the time*’; 5 *‘Always’*	Students whose parents most of the time/always respect their children or their personal space (reverse scale) *(Yes vs. No)*
Have close friends	How many close friends do you have? *1 ‘0’ to 3 ‘3 or more’*	Students who had any close friends *(Yes vs. No)*
Loneliness	During the past 12 months, how often have you felt lonely? 1 *‘Never’*; 2 *‘Rarely’*; 3 *‘Sometimes’*; 4 *‘Most of the time*’; 5 *‘Always’*	Students who most of the time/always felt lonely during the past 12 months *(Yes vs. No)*
Suicidal ideation	During the past 12 months, did you ever seriously consider attempting suicide? *1 ‘No’; 2 ‘Yes’*	Students who seriously considered attempting suicide in the past 12 months *(Yes vs. No)*
Violence	During the past 12 months, how many times were you physically attacked? 1 *‘0 time*’; 2 *‘1 time’ to* 8 *‘12 or more time’*	Students who were physically attacked in the past 12 months *(Yes vs. No)*
Self-reported experiences of bullying	During the past 30 days, on how many days were you bullied at school (or near school or on the way to and from school)? *1 ‘0 day’ to 7 ‘all 30 days’*	Students who were bullied at school in the past 30 days *(Yes vs. No)*
Sexual activity	Have you ever had sexual intercourse? 0 *‘No*’; 1 *‘Yes’*	Students who have ever had sexual intercourse *(Yes vs. No)*
Truancy	During the past 30 days, on how many days did you miss classes or school without permission? *1 ‘0 day’ to 5 ‘10 days or more’*	Students who missed any days without permission during the past 30 days *(Yes vs. No)*
Alcohol consumption	During the past 30 days, on how many days did you have at least one drink containing alcohol? 1 ‘0 days’ to 7 ‘All 30 days’	Students who drank at least 1 drink in the past 30 days *(Yes vs. No)*
Sedentary lifestyle	How much time do you spend during a typical or usual day sitting and watching television, playing computer games, relaxing with iPad, mobile phone, talking with friends, or doing other sitting activities, such as book reading or using Facebook? 1 *‘Less than 1 hour per day*’; 2 *‘1 to 2 hours per day’*; 3 *‘3 to 4 hours per day’* to 6 *‘More than 8 hours per day’*	Student who spent three or more hours a day to do these activities *(Yes vs. No)*
Low fruit/vegetable intake	- During the past 30 days, on average how many times per day did you usually eat fruit, such as a banana, apple, orange, guava, rambutan, watermelon, papaya, or mango etc.? - During the past 30 days, on average how many times per day did you usually eat vegetables, such as morning glory, cabbage, or mustard green etc.? *1 ‘I did not eat’ to 7 ‘5 or more times per day’*	Students who ate less than 5-time fruits/vegetables per day in the past 30 days *(Yes vs. No)*
People smoked in presence in the past 7 days	During the past 7 days, on how many days have people smoked in your presence (at least one time per day)? *1 ‘0 days’ to 5 ‘All 7 days’*	Students who had seen/contacted with smokers in the past 7 days *(Yes vs. No)*
Smoked Shisha in the past 30 days	During the past 30 days, on how many days did you use Shisha? *1 ‘0 days’ to 7 ‘All 30 days’*	Students who smoked shisha at least 1 time in the past 30 days *(Yes vs. No)*

### Data processing and analysis

A weighting factor was applied to each in-school adolescent record to reflect the likelihood of sampling each in-school adolescent and reduce bias by compensating for different patterns of non-response. The weight used for the estimation is given as follows:

W = W1 ∗ W2 ∗ W3 ∗ f1 ∗ f2 ∗ f3

In which:

W1 is the inverse of the probability of selecting the province.

W2 is the inverse of the probability of selecting the school.

W3 is the inverse of the probability of selecting the classroom within the school.

f1 is a school-level non-response adjustment factor calculated by the school.

f2 is an in-school adolescent-level non-response adjustment factor calculated by the class size category. This factor was calculated in terms of class enrolment, instead of the number of classes.

f3 is a post-stratification adjustment factor calculated using rural/urban and grade.

In the 2019 GSHS, out of 7,796 respondents, 106 self-identified as ‘other-gender’. Due to the small sample size of this group and in order to maintain consistency with the 2013 GSHS, we only included male and female in-school adolescents (n = 7,690) in the analysis. Multiple logistic regression was used to identify factors related to current and e-cigarette smoking. The variables included in the models were based on prior evidence of the factors related to the smoking status of adolescents [12,15,16]. These models included only male respondents, as a negligible number of female in-school adolescents reported being smokers. All analyses were conducted using Stata v16 with the survey command ‘*svy’* to account for the complex sample design (strata, cluster, and weights).

## Results

### Characteristics of the study on in-school adolescents

The characteristics of the study participants are presented in Table 2. The patterns of parental monitoring, having close friends, reporting loneliness, attempting suicide, engaging in sexual activity, and living a sedentary lifestyle were similar in both surveys. The percentage of in-school adolescents who reported being understood and respected by their parents was higher in the 2019 GSHS. The proportion of in-school adolescents who experienced violence, bullying, truancy, and alcohol consumption in the prior 30 days was lower in the 2019 GSHS.

**Table 2.** Participants’ characteristics.

Characteristics	2013		2019	
	n	Weighted %	n	Weighted %
**N**	**3331**		**7690**	
**Gender**				
Male	1,557	46.9	3,572	46.0
Female	1,765	53.1	4,118	54.0
**Age**				
13	897	21.7	1,024	16.7
14	858	22.5	1,523	30.4
15	542	19.6	1,672	19.9
16	769	23.9	1,646	15.9
17	264	12.3	1,825	17.1
**Parental monitoring**				
Low	1,664	52.2	4,252	52.3
High	1,662	47.8	3,410	47.7
**Parental understanding**				
Low	2,283	69.5	3,586	44.2
High	1,038	30.5	4,102	55.8
**Parental respect**				
Low	1,209	36.8	1,176	14.3
High	2,070	63.2	6,451	85.7
**Have close friends**				
No	173	5.5	778	8.8
Yes	3,138	94.5	6,816	91.2
**Loneliness**				
No	2,910	88.5	6,589	87.7
Yes	367	11.5	1,098	12.3
**Suicidal ideation**				
No	2,750	83.1	6,333	84.6
Yes	539	16.9	1,347	15.4
**Violence**				
No	2,582	79.0	6,885	89.6
Yes	734	21.0	802	10.4
**Bullied**				
No	2,456	77.3	7,258	94.5
Yes	744	22.7	427	5.5
**Sexual activity**				
No	3,002	93.5	7,188	94.9
Yes	187	6.5	459	5.1
**Truancy**				
No	2,725	80.6	6,307	84.9
Yes	598	19.4	1,191	15.1
**Alcohol consumption in the past 30 days**				
No	2,476	75.1	5,781	77.9
Yes	722	24.9	1,864	22.1
**Sedentary lifestyle**				
No	1,968	58.0	3,942	57.1
Yes	1,349	42.0	3,683	42.9
**Low fruit/vegetable intake**				
No	693	20.5	370	4.6
Yes	2,603	79.5	7,295	95.4

### The pattern of current tobacco smoking among in-school adolescents

As shown in Table 3, the prevalence of current tobacco smoking (both water pipes and cigarettes) among in-school adolescents in 2013 and 2019 was 5.4% (95% CI: 4.0–7.2) and 2.8% (95% CI: 2.2–3.6), respectively. The difference was statistically significant as the 95% CI did not overlap. Significant decreases in the prevalence of smoking among in-school adolescents from 2013 to 2019 were consistent for both cigarette and water pipe use. In 2019, 2.6% of in-school adolescents reported using electronic cigarette products in the past 30 days.

**Table 3.** Smoking prevalence and trend from 2013 to 2019.

Characteristics	2013		2019	
	Weighted %	95% CI	Weighted %	95% CI
**All**				
Have ever smoked	12.1	9.8–15.0	8.2	7.0–9.6
Smoked cigarettes in the past 30 days	4.7	3.5–6.3	2.6	2.0–3.4
Smoked water pipe in the past 30 days	2.4	1.5–3.8	1.0	0.7–1.4
Traditional tobacco smoking (cigarettes and water pipe) in the past 30 days	5.4	4.0–7.2	2.8	2.2–3.6
People smoked in presence in the past 7 days	75.8	73.4–78.0	66.0	63.8–68.2
Smoked Shisha in the past 30 days	N/A	N/A	1.3	0.93–1.72
Smoked e–cigarettes in the past 30 days	N/A	N/A	2.6	1.9–3.3
**Males**				
Have ever smoked	19.8	16.8–23.3	13.3	11.0–15.9
Smoked cigarettes in the past 30 days	8.8	6.3–12.1	4.4	3.2–6.1
Smoked water pipe in the past 30 days	4.2	2.6–6.5	1.9	1.4–2.8
Traditional tobacco smoking (cigarettes and water pipe) in the past 30 days	9.6	6.9–13.2	4.9	3.7–6.5
People smoked in presence in the past 7 days	75.7	72.2–78.8	65.0	62.2–67.7
Smoked Shisha in the past 30 days	N/A	N/A	1.7	1.0–2.4
Smoked e–cigarettes in the past 30 days	N/A	N/A	3.6	2.6–4.7
**Females**				
Have ever smoked	5.5	3.5–8.5	4.0	3.0–5.2
Smoked cigarettes in the past 30 days	1.1	0.6–2.1	1.0	0.7–1.5
Smoked water pipe in the past 30 days	0.8	0.4–1.9	0.2	0.1–0.5
Traditional tobacco smoking (cigarettes and water pipe) in the past 30 days	1.7	1.0–2.8	1.0	0.7–1.5
People smoked in presence in the past 7 days	76.1	73.0–78.9	66.9	64.6–69.1
Smoked Shisha in the past 30 days	N/A	N/A	1	0.6–1.4
Smoked e–cigarettes in the past 30 days	N/A	N/A	1.5	0.8–2.3

The prevalence of current traditional tobacco smoking and e-cigarette smoking among in-school adolescents according to their characteristics (among all in-school adolescents and male in-school adolescent groups) is shown in Tables S1 and S2.

### Factors associated with traditional tobacco smoking among in-school adolescents

Table 4 shows the results of the logistic regression analyses of factors associated with traditional tobacco smoking status among in-school adolescents. After controlling for other independent variables in the model, the statistically significant factors associated with current smoking status were as follows: 1) year of data collection: lower in 2019 (OR = 0.66); 2) gender: higher among male in-school adolescents (OR = 4.25); 3) parental monitoring: lower in the group with high levels of parental monitoring (OR = 0.57); 4) loneliness: higher among those who felt loneliness (OR = 1.57); 5) suicidal ideation: higher among those who had suicidal ideation (OR = 1.71); 6) sexual intercourse: higher among those who had sexual intercourse (OR = 4.56); 7) truancy: higher among those who reported truancy (OR = 2.23); and 8) alcohol drinking: higher among those who drank alcohol in the past 30 days (OR = 4.17). Among male in-school adolescents, after controlling for other independent variables in the model, a statistically significant association was found with age, with higher odds of tobacco smoking among older in-school adolescents (OR = 2.0–2.3). All associated factors found in the model remained significant for males, except for study year, loneliness, and suicidal ideation.

**Table 4.** Factors related to traditional tobacco smoking among students aged 13–17 in Vietnam.

Factor	All students		Male students	
	OR	95% CI	OR	95% CI
**Year of data collection**, *(Ref: 2013)*				
2019	0.66*	**0.44–0.99**	0.63	0.39–1.00
**Gender**, *(Ref: Female)*				
Male	4.25***	**2.56–7.04**	**–**	**–**
**Age**, *(Ref: 13)*				
14	1.19	0.71–2.00	1.40	0.63–3.11
15	1.27	0.85–1.92	2.00*	**1.16–3.44**
16	1.41	0.86–2.30	2.30**	**1.24–4.27**
17	1.45	0.93–2.25	2.30*	**1.18–4.48**
**Parental monitoring**, *(Ref: Low)*				
High	0.57*	**0.37–0.88**	0.58*	**0.34–0.97**
**Parental understanding**, *(Ref: Low)*				
High	0.90	0.63–1.30	0.97	0.62–1.51
**Parental respect**, *(Ref: Low)*				
High	0.73	0.51–1.04	0.84	0.57–1.24
**Have close friends**, *(Ref: No)*				
Yes	1.28	0.73–2.23	1.19	0.61–2.31
**Loneliness**, *(Ref: No)*				
Yes	1.57*	**1.10–2.25**	1.37	0.82–2.26
**Suicidal ideation**, *(Ref: No)*				
Yes	1.71*	**1.10–2.67**	1.61	0.84–3.06
**Violence**, *(Ref: No)*				
Yes	1.31	0.80–2.17	1.36	0.77–2.38
**Bullied**, *(Ref: No)*				
Yes	0.82	0.50–1.33	0.89	0.50–1.59
**Sexual intercourse**, *(Ref: No)*				
Yes	4.56***	**3.17–6.57**	4.57***	**3.13–6.66**
**Truancy**, *(Ref: No)*				
Yes	2.23**	**1.38–3.61**	2.31**	**1.29–4.14**
**Alcohol consumption in the past 30 days**, *(Ref: No)*				
Yes	4.17***	**2.78–6.24**	3.79***	**2.31–6.22**
**Sedentary lifestyle**, *(Ref: No)*				
Yes	0.98	0.71–1.36	0.86	0.61–1.22
**Low fruit/vegetable intake**, *(Ref: No)*				
Yes	1.11	0.73–1.67	1.28	0.71–2.31

* *p* < 0.05; ** *p* < 0.01; *** *p* < 0.001.

### Factors associated with e-cigarette smoking among in-school adolescents

The factors associated with e-cigarette use among in-school adolescents in 2019 are shown in Table 5. We found that being male, having attempted suicide in the past twelve months, engaging in sexual activity, being bullied, truancy, drinking alcohol, and spending three or more hours in a sedentary lifestyle were significant factors in increasing the odds of e-cigarette use. Specifically, in-school adolescents who reported drinking alcohol in the past 30 days were five times more likely to be e-cigarette users (OR = 5.38, 95% CI: 3.75–7.71). However, high levels of parental monitoring and respect decreased the odds of being e-cigarette users by 44% and 36% among in-school adolescents, respectively. All these associated factors remained significant in the regression model for male in-school adolescents, except for parental respect and truancy. Age, parental understanding, loneliness, trauma, and fruit and vegetable intake were not significantly associated with e-cigarette use among in-school adolescents.

**Table 5.** Factors related to e-cigarettes smoking among students aged 13–17 in Vietnam.

Factor	All students		Male students	
	OR	95% CI	OR	95% CI
**Gender**, *(Ref: Female)*				
Male	2.09**	**1.28–3.42**		
**Age**, *(Ref: 13)*				
14	0.56	0.22–1.44	0.57	0.19–1.72
15	0.46	0.17–1.25	0.67	0.21–2.13
16	0.42	0.14–1.23	0.56	0.18–1.73
17	0.64	0.21–2.00	1.08	0.33–3.50
**Parental monitoring**, *(Ref: Low)*				
High	0.56**	**0.40–0.78**	0.54**	**0.35–0.84**
**Parental understanding**, *(Ref: Low)*				
High	1.22	0.82–1.81	1.16	0.75–1.82
**Parental respect**, *(Ref: Low)*				
High	0.64*	**0.43–0.97**	0.67	0.36–1.24
**Have close friends**, *(Ref: No)*				
Yes	1.20	0.68–2.12	1.08	0.48–2.40
**Loneliness**, *(Ref: No)*				
Yes	1.08	0.70–1.68	0.93	0.48–1.83
**Suicidal ideation**, *(Ref: No)*				
Yes	1.75*	**1.08–2.83**	1.49	0.83–2.68
**Violence**, *(Ref: No)*				
Yes	1.20	0.55–2.59	1.06	0.48–2.34
**Bullied**, *(Ref: No)*				
Yes	2.67**	**1.35–5.27**	3.38**	**1.69–6.79**
**Sexual intercourse**, *(Ref: No)*				
Yes	3.05***	**1.71–5.44**	2.97**	**1.46–6.03**
**Truancy**, *(Ref: No)*				
Yes	1.68*	**1.11–2.54**	1.01	0.64–1.59
**Alcohol consumption in the past 30 days**, *(Ref: No)*				
Yes	5.38***	**3.75–7.71**	3.37***	**2.27–5.00**
**Sedentary lifestyle**, *(Ref: No)*				
Yes	1.81**	**1.25–2.60**	1.54*	**1.05–2.27**
**Low fruit/vegetable intake**, *(Ref: No)*				
Yes	0.66	0.30–1.47	1.01	0.33–3.08

* *p* < 0.05; ** *p* < 0.01; *** *p* < 0.001.

## Discussion

Tobacco smoking is one of the most dangerous risk behaviours to an individual’s health. Monitoring tobacco use is an important component of the WHO Framework Convention on Tobacco Control (WHO FCTC) and MPOWER (a package of selected demand reduction measures contained in the WHO FCTC) [17]. In Vietnam, MPOWER has been implemented since 2008; however, most of the studies focused on the adult population. The data on smoking in adolescents are sparse, especially for e-cigarette use. This is a recent trend for which there are little data [1]. The problem of adolescent smoking in Vietnam became a top priority in the tobacco control policies in Vietnam because the tobacco industry has been trying to expand tobacco markets by promoting cigarettes to adolescents. Scientific evidence on the prevalence of smoking, including traditional tobacco and e-cigarettes, among in-school adolescents and related factors is needed to identify high-risk groups and potential protective factors to inform prevention programmes. The GSHS in 2013 and 2019, are important data sources for evaluating and adjusting tobacco control policies in Vietnam focusing on children and adolescents.

In this study, data from the Vietnam 2013 and 2019 GSHS were used to describe the prevalence of tobacco smoking and associated factors among in-school adolescents aged 13–17 years old. The numbers (and percentages) of consent forms that were not returned by parents in 2013 and 2019 were 139 (4%) and 542 (6.5%), respectively. The prevalence of traditional tobacco smoking decreased significantly between 2013 and 2019. The prevalence of e-cigarette use among in-school adolescents 30 days prior was 2.6% in 2019. The factors associated with higher odds of traditional smoking included gender, mental health problems, sexual activity, truancy, and alcohol consumption. Risk factors for e-cigarette smoking included gender, mental health problems, self-reported experience of bullying, sexual activity, truancy, alcohol consumption, and sedentary lifestyle. In contrast, the protective factor for traditional smoking was parental monitoring, while those for e-cigarette smoking were both parental monitoring and parental respect.

From 2013 to 2019, we observed a significant decrease in the prevalence of tobacco smoking among in-school adolescents. The Global Adult Tobacco Use Survey (GATS 2015) in Vietnam also indicated a decreasing trend in adult smoking rates from 23.8% in 2010 to 22.5% in 2015 [18]. This trend can be explained by the implementation of active tobacco control in Vietnam. Following the establishment of the Vietnam Tobacco Control Law, tobacco advertising and promotion have been prohibited in the country. Pictorial health warnings must be printed on cigarette packages to warn people about the dangers of tobacco. Tobacco-related risk communication activities have been effectively implemented to receive attention and increase population awareness. However, Vietnam is still facing tobacco control issues regarding the level of cigarette taxes and the availability of tobacco cessation services. The cigarette tax level is now only approximately 75% of the factory price, which is less than 39% of the retail price [19], and is much lower than the threshold level of 70% of the retail price recommended by WHO [20]. The price of a pack of 20 cigarettes of the most popular brand in Vietnam was only $US 0.91 in 2017 [19]. However, tobacco cessation services are limited. Concern over tobacco use in healthcare is still limited; only 34.9% of the smokers were asked about their history of tobacco use and less than one-third of them (29.7%) received advice to quit smoking [21].

In the literature, the prevalence of tobacco smoking varies across countries and time periods surveyed. According to a multi-country survey of tobacco use among adolescents aged 13–15 years (GSHS data from 44 countries, 110 sites) [22], the overall prevalence of tobacco use ranged from 0.9% in Tajikistan (2006) to 32.8% in Chile (2005). In Southeast Asia, the prevalence of tobacco smoking among in-school adolescents ranged from 4.5% in Myanmar in 2007 [23] to 12.6% in Indonesia in 2012 [23]. Within the Western Pacific Region, this prevalence ranged from 4.1% in Cambodia (2013) [23] to 21.1% in the Cook Islands (2011) [23].

Several studies have reported a worldwide declining trend in tobacco use among adolescents. Choi et al. used data from the Health and Nutrition Examination Survey from 1998 to 2013, and the Korea Youth Risk Behaviour Web-based Survey from 2005 to 2013 indicated a clear declining trend among girls [24]. A global report on the trends of tobacco use from 2000 to 2025 showed that smoking prevalence among people aged 15–24 years will decrease globally from 22.6% in 2010 to 14.2% in 2025 [5].

Consistent with previous studies, our study confirmed that the risk factors for tobacco smoking include male gender [15,24,25], mental health problems [16,26], sexual activity [27], truancy [28,29], and alcohol consumption [15,25,28].

The prevalence of e-cigarette uses among youths varies widely across countries. For example, the reported prevalence of daily use of e-cigarettes among New Zealander youth aged 14–15 years in 2019 was 3.1% in 2019 [30], only 4.4% of adolescents aged 15–17 years in Taiwan reported ever having used e-cigarettes in 2015 [31], and 27.5% of high-school students and 10.5% of middle school students in the US reported current e-cigarette use (30 days prior) in 2019 [32]. In addition, the prevalence of e-cigarette use among the youth has increased rapidly over a short period in some countries [33]. E-cigarette use among US high-school students, for instance, rose from 1.5% to 16% during the 2011–2015 period [34] before reaching the current level. The prevalence of e-cigarette use among Vietnamese in-school adolescents in our study in 2019 (2.6%) appears lower than that in the aforementioned countries. This can be explained by the current ban on e-cigarette importation into Vietnam. All e-cigarette products in the market are illegal/smuggled products.

Most e-cigarettes contain nicotine, which can lead to respiratory ailments and have negative impacts on attention, learning, and memory among adolescents and young adults, while the long-term health effects of e-cigarette use remain unknown [35–37]. Moreover, some evidence indicates that e-cigarette use in adolescents can be a gateway to the initiation of illicit drugs and other tobacco products [36,38], thereby causing more harmful effects. The design of e-cigarette devices and the variety of flavoured solutions (e.g. menthol, fruit, coffee, candy, etc.) that hide the harsh taste of nicotine can appeal to adolescents. Another concern is that many adolescents and young adults may perceive vaping as trendy, socially acceptable, and/or having no harmful effects [39,40]. Additionally, adolescents are susceptible to peer influences, which can make e-cigarette use more popular. Therefore, despite the low prevalence of e-cigarette smoking among adolescents in Vietnam, adequate attention should be paid to tobacco control experts. We also found that e-cigarette use was associated with other unhealthy behaviours among Vietnamese in-school adolescents, such as alcohol drinking, engaging in unprotected sexual activity, truancy, and sedentary behaviours. This suggests that health education programmes for adolescents should comprehensively target these compound risk factors rather than just one individual factor. However, longitudinal studies are required to determine the causal factors and pathways associated with e-cigarette use among Vietnamese adolescents.

A high level of parental monitoring is associated with lower odds of smoking among in-school adolescents. Because Vietnam has a traditional family-oriented culture, parental factors always play a crucial role in children’s behaviours. Some studies have also shown the protective effects of parental factors on children’s negative behaviours, including smoking frequency. Castrucci’s research on the association between parenting style and adolescent smoking in the US indicated that parenting style was associated with a 26% reduction in adolescent smoking. A study in the Cook Islands, Curacao, and East Timor also found that parental monitoring, which was measured in four aspects (understanding, knowing free time activities of children, going over things without permission, and checking homework), was associated with lower odds of smoking in adolescents aged 13–17 years old [41]. Together, the results highlight the need to incorporate parental involvement in programmes designed to reduce tobacco smoking among adolescents.

### Limitations

This study has several limitations. First, despite a nationally representative school-based sampling frame, the results from this study cannot be generalised to all Vietnamese adolescents, as those who were not attending school were not included in this study. In addition, owing to the cross-sectional design, we were unable to determine causal factors for the decreasing trend in cigarette smoking, as well as those for the emergence of e-cigarette use among Vietnamese in-school adolescents. Third, the data were self-reported and might have been subject to recall and response bias. Fourth, the data were collected only among participants who returned the consent form from their parents or guardians. This could have introduced selection bias.

## Conclusion

The overall prevalence of smoking among in-school adolescents in Vietnam decreased between 2013 and 2019. Smoking prevalence was significantly higher among males than females. More attention is needed to further reduce smoking behaviour among in-school adolescents, especially among those who have other behavioural risk factors, including mental health problems, frequent sexual activity, truancy, exposure to bullying, and alcohol consumption.

## Supplementary Material

Supplemental Material

Supplemental Material

## Paper context

This study investigated the trend in tobacco smoking between 2013 and 2019, risk factors for tobacco smoking and e-cigarette use among Vietnamese in-school adolescents. The trend decreased from 5.4% to 2.8%. The e-cigarette use prevalence increased from 0% in 2013 to 2.6% in 2019. Higher tobacco and e-cigarette smoking status was associated with gender, loneliness, suicidal ideation, sexual activity, truancy, and alcohol consumption. The results demonstrated the need to develop policies targeting this issue.

## Preprint citation

Minh HV, Long KQ, Vuong DV, Hung NM, Park K, Takeuchi M Tobacco smoking and its associated factors among in-school adolescents in Vietnam in 2013 and 2019. medRxiv. 2021. (Available at https://doi.org/10.1101/2021.03.28.21254501)
