# Supplementary material for: Tobacco and electronic cigarette smoking among in-school adolescents in Vietnam between 2013 and 2019: prevalence and associated factors
Source: Glob Health Action. 2022 Sep 29;15(1):2114616. doi: 10.1080/16549716.2022.2114616 (PMC9542268; doi:10.1080/16549716.2022.2114616)
Supplement: Supplemental Material [file ZGHA_A_2114616_SM3204.docx]

**Supplemental Table 1**

*Prevalence of traditional tobacco smoking by students’ characteristics*

| **Factor** | **All students** | | | | **Male students** | | | |
| --- | --- | --- | --- | --- | --- | --- | --- | --- |
|  | **2013** | | **2019** | | **2013** | | **2019** | |
|  | **%** | **95% CI** | **%** | **95% CI** | **%** | **95% CI** | **%** | **95% CI** |
| **Gender** |  |  |  |  |  |  |  |  |
| Male | 9.6 | 6.8–13.3 | 4.9 | 3.6–6.5 | -- | -- | -- | -- |
| Female | 1.7 | 0.9–2.9 | 1.0 | 0.7–1.5 | -- | -- | -- | -- |
| **Age** |  |  |  |  |  |  |  |  |
| 13 | 2.7 | 1.9–3.8 | 1.9 | 1.2–2.9 | 4.0 | 2.6-6.0 | 2.8 | 1.5-5.0 |
| 14 | 3.3 | 2.4–4.5 | 2.8 | 1.8–4.5 | 5.0 | 3.5-6.9 | 5.0 | 2.6-9.2 |
| 15 | 6.2 | 4.7–8.2 | 2.0 | 1.1–3.5 | 11.0 | 8.2-14.5 | 3.8 | 2.2-6.5 |
| 16 | 7.0 | 4.0–12.2 | 2.6 | 1.7–3.9 | 13.3 | 8.0-21.3 | 4.3 | 2.9-6.2 |
| 17 | 9.2 | 5.0–16.5 | 4.6 | 3.3–6.5 | 18.4 | 9.2-33.4 | 8.4 | 5.7-12.3 |
| **Parental monitoring** |  |  |  |  |  |  |  |  |
| Low | 7.2 | 4.9–10.4 | 3.3 | 2.4–4.5 | 13.1 | 8.3-20.0 | 6.0 | 4.1-8.8 |
| High | 3.3 | 2.3–4.9 | 2.1 | 1.4–3.2 | 6.1 | 4.1-9.1 | 3.6 | 2.5-5.3 |
| **Parental understanding** |  |  |  |  |  |  |  |  |
| Low | 5.9 | 4.1–8.3 | 3.1 | 2.3–4.3 | 10.6 | 7.1-15.5 | 5.1 | 3.3-7.8 |
| High | 4.1 | 2.9–5.9 | 2.5 | 1.7–3.7 | 7.4 | 5.1-10.7 | 4.7 | 3.2-7.0 |
| **Parental respect** |  |  |  |  |  |  |  |  |
| Low | 7.0 | 4.6–10.4 | 5.8 | 4.1-8.2 | 11.2 | 6.8-18.0 | 8.8 | 5.9-13.0 |
| High | 4.2 | 3.0–5.8 | 2.2 | 1.6-3.0 | 8.0 | 5.7-11.2 | 4.1 | 2.8-5.8 |
| **Have close friends** |  |  |  |  |  |  |  |  |
| No | 8.0 | 4.4–13.9 | 3.0 | 1.9–4.8 | 12.3 | 6.6-21.9 | 5.5 | 3.5-8.7 |
| Yes | 5.1 | 3.7–7.1 | 2.7 | 2.1–3.5 | 9.2 | 6.3-13.4 | 4.7 | 3.4-6.4 |
| **Loneliness** |  |  |  |  |  |  |  |  |
| No | 4.6 | 3.2–6.6 | 2.4 | 1.9–3.2 | 8.5 | 5.8-12.5 | 4.3 | 3.2-5.8 |
| Yes | 11.2 | 7.9–15.6 | 5.2 | 3.6–7.4 | 18.5 | 12.5-26.6 | 9.0 | 6.2-13.1 |
| **Suicidal attempt** |  |  |  |  |  |  |  |  |
| No | 4.4 | 2.8–6.8 | 2.4 | 1.8–3.1 | 7.8 | 4.9-12.4 | 4.4 | 3.2-5.9 |
| Yes | 9.4 | 6.8–12.7 | 4.7 | 3.3–6.7 | 19.9 | 13.4-28.5 | 8.3 | 5.3-12.6 |
| **Violence** |  |  |  |  |  |  |  |  |
| No | 4.7 | 3.1–7.1 | 2.1 | 1.6–2.8 | 8.8 | 5.6-13.6 | 3.9 | 2.9-5.3 |
| Yes | 7.8 | 6.1–9.9 | 8.4 | 5.8–11.9 | 12.0 | 9.2-15.5 | 10.1 | 7.1-14.0 |
| **Bullied** |  |  |  |  |  |  |  |  |
| No | 5.0 | 3.3–7.7 | 2.5 | 1.9–3.3 | 8.6 | 5.3-13.7 | 4.5 | 3.3-6.1 |
| Yes | 6.0 | 4.5–8.0 | 7.7 | 4.7–12.4 | 12.0 | 8.9-16.0 | 10.3 | 6.2-16.8 |
| **Sexual intercourse** |  |  |  |  |  |  |  |  |
| No | 3.6 | 2.7–4.7 | 2.2 | 1.7–2.9 | 6.3 | 4.5-8.8 | 3.9 | 2.8-5.4 |
| Yes | 25.4 | 17.0–36.3 | 12.2 | 8.4–17.6 | 36.7 | 25.6-49.5 | 17.1 | 11.5-24.8 |
| **Truancy** |  |  |  |  |  |  |  |  |
| No | 3.4 | 2.5–4.6 | 2.0 | 1.4–2.7 | 5.9 | 4.2-8.4 | 3.7 | 2.5-5.5 |
| Yes | 13.4 | 9.3–18.7 | 6.9 | 5.5–8.8 | 21.0 | 14.2-29.8 | 9.5 | 7.0-12.9 |
| **Drinking alcohol in the past 30 days** |  |  |  |  |  |  |  |  |
| No | 2.0 | 1.3–3.0 | 1.2 | 0.8–1.7 | 3.8 | 2.3-6.3 | 2.2 | 1.5-3.3 |
| Yes | 15.5 | 11.8–20.1 | 8.3 | 6.4–10.9 | 21.3 | 15.9-27.9 | 13.0 | 9.3-17.7 |
| **Sedentary lifestyles** |  |  |  |  |  |  |  |  |
| No | 4.3 | 3.1–6.1 | 2.5 | 1.7–3.7 | 7.9 | 5.3-11.6 | 4.6 | 3.1-6.7 |
| Yes | 6.4 | 4.7–8.8 | 3.2 | 2.4–4.3 | 11.2 | 8.3-15.0 | 5.4 | 3.7-7.9 |
| **Low fruit/vegetables intake** |  |  |  |  |  |  |  |  |
| No | 5.5 | 3.1–9.4 | 4.2 | 2.0–8.6 | 10.0 | 5.0-18.9 | 4.7 | 2.3-9.4 |
| Yes | 5.3 | 3.9-7.2 | 2.7 | 2.1-3.5 | 9.5 | 6.9-13.0 | 4.9 | 3.6-6.5 |

**Supplemental Table 2**

*Prevalence of e-cigarettes smoking by students’ characteristics*

| **Factor** | **All students** | | **Male students** | |
| --- | --- | --- | --- | --- |
|  | **%** | **95% CI** | **%** | **95% CI** |
| **Gender** |  |  |  |  |
| Male | 3.6 | 2.7-4.8 | -- | -- |
| Female | 1.5 | 0.9-2.5 | -- | -- |
| **Age** |  |  |  |  |
| 13 | 3.1 | 1.5-6.2 | 3.8 | 1.5-9.1 |
| 14 | 1.8 | 1.0-3.0 | 2.1 | 1.2-3.5 |
| 15 | 1.9 | 1.3-3.0 | 3.0 | 1.8-4.9 |
| 16 | 2.5 | 1.6-3.8 | 3.6 | 2.3-5.5 |
| 17 | 3.9 | 2.4-6.2 | 6.7 | 4.1-10.8 |
| **Parental monitoring** |  |  |  |  |
| Low | 3.2 | 2.4-4.3 | 4.9 | 3.4-6.9 |
| High | 1.7 | 1.2-2.4 | 2.5 | 1.8-3.5 |
| **Parental understanding** |  |  |  |  |
| Low | 2.8 | 2.1-3.8 | 4.1 | 2.9-5.7 |
| High | 2.2 | 1.5-3.2 | 3.3 | 2.3-4.8 |
| **Parental respect** |  |  |  |  |
| Low | 5.1 | 3.4-7.7 | 6.5 | 3.9-10.6 |
| High | 2.1 | 1.5-2.8 | 3.1 | 2.2-4.3 |
| **Have close friends** |  |  |  |  |
| No | 3.4 | 2.1-5.5 | 4.8 | 2.6-8.7 |
| Yes | 2.4 | 1.8-3.3 | 3.5 | 2.6-4.7 |
| **Loneliness** |  |  |  |  |
| No | 2.1 | 1.5-3.0 | 3.2 | 2.3-4.5 |
| Yes | 5.0 | 3.5-7.1 | 6.7 | 4.4-10.2 |
| **Suicidal attempt** |  |  |  |  |
| No | 2.0 | 1.4-2.7 | 3.1 | 2.2-4.2 |
| Yes | 5.3 | 3.6-7.7 | 7.4 | 4.7-11.5 |
| **Violence** |  |  |  |  |
| No | 2.1 | 1.4-3.0 | 3.2 | 2.3-4.3 |
| Yes | 6.2 | 4.5-8.5 | 6.2 | 4.2-9.0 |
| **Bullied** |  |  |  |  |
| No | 2.1 | 1.5-2.9 | 2.9 | 2.1-4.1 |
| Yes | 10.1 | 6.7-14.9 | 13.3 | 8.7-19.7 |
| **Sexual intercourse** |  |  |  |  |
| No | 2.1 | 1.5-2.8 | 2.9 | 2.2-4.0 |
| Yes | 10.6 | 6.6-16.7 | 13.2 | 7.6-21.9 |
| **Truancy** |  |  |  |  |
| No | 1.9 | 1.4-2.6 | 3.1 | 2.3-4.2 |
| Yes | 5.5 | 3.6-8.2 | 5.4 | 3.4-8.3 |
| **Drinking alcohol in the past 30 days** |  |  |  |  |
| No | 1.0 | 0.7-1.5 | 1.9 | 1.3-2.8 |
| Yes | 7.5 | 5.5-10.2 | 8.9 | 6.5-12.1 |
| **Sedentary lifestyles** |  |  |  |  |
| No | 1.6 | 1.2-2.2 | 2.5 | 1.9-3.5 |
| Yes | 3.7 | 2.7-5.0 | 5.4 | 3.8-7.6 |
| **Low fruit/vegetables intake** |  |  |  |  |
| No | 3.7 | 1.8-7.1 | 3.1 | 1.1-8.4 |
| Yes | 2.4 | 1.8-3.3 | 3.7 | 2.7-4.9 |
