# Supplementary material for: Tobacco and electronic cigarette smoking among in-school adolescents in Vietnam between 2013 and 2019: prevalence and associated factors
Source: Glob Health Action. 2022 Sep 29;15(1):2114616. doi: 10.1080/16549716.2022.2114616 (PMC9542268; doi:10.1080/16549716.2022.2114616)
Supplement: Supplemental Material [file ZGHA_A_2114616_SM3078.docx]

**Table 1**

*Definitions of dependent and independent variables analyzed*

| **Variables** | **Question** | **Definition used in this paper** |
| --- | --- | --- |
| Dependent variables | | |
| Smoked traditional tobacco | - During the past 30 days, on how many days did you smoke cigarettes?  - During the past 30 days, on how many days did you use “thuoc lao” (Vietnamese water pipe)?  *1 “0 days” to 7 “All 30 days”* | Students who smoked at least 1 time (cigarettes or Vietnamese water pipe) in the past 30 days  *(Yes vs. No)* |
| Smoked e-cigarettes | During past 30 days, on how many days did you use electronic cigarettes?  *1 “0 days” to 7 “All 30 days”* | Students who smoked e-cigarettes at least 1 time in the past 30 days  *(Yes vs. No)* |
| Independent variables | | |
| Gender |  | Male vs. Female |
| Age | How old are you?  1 *“13 year-old” to* 5 *“17 year-old or older”* | From 13 to 17 |
| Parental monitoring | - During the past 30 days, how often did your parents or guardians check to see if your homework was done?  - During the past 30 days, how often did your parents or guardians really know what you were doing with your free time?  1 *“Never”*; 2 *“Rarely”*; 3 *“Sometimes”*; 4 *“Most of the time”;* 5 *“Always”* | Students whose parents most of the time/always checked homework or know what their children do in their free time  *(High vs. Low)* |
| Parental understanding | - During the past 30 days, how often did your parents or guardians understand your problems and worries?  - During the past 30 days, how often did your parents or guardians give you advice and guidance?  1 *“Never”*; 2 *“Rarely”*; 3 *“Sometimes”*; 4 *“Most of the time”;* 5 *“Always”* | Students whose parents most of the time/always understood problems/worries or gave advice/guidance  *(High vs. Low)* |
| Parental respect | - During the past 30 days, how often did your parents or guardians go through your things without your approval?  - During the past 30 days, how often did your parents or guardians not respect you as a person (for example, not let you talk or favor someone else more than you)?  1 *“Never”*; 2 *“Rarely”*; 3 *“Sometimes”*; 4 *“Most of the time”;* 5 *“Always”* | Students whose parents most of the time/always respect their children or their personal space (reverse scale)  *(Yes vs. No)* |
| Have close friends | How many close friends do you have?  *1 “0” to 3 “3 or more”* | Students who had any close friends  *(Yes vs. No)* |
| Loneliness | During the past 12 months, how often have you felt lonely?  1 *“Never”*; 2 *“Rarely”*; 3 *“Sometimes”*; 4 *“Most of the time”;* 5 *“Always”* | Students who most of the time/always felt lonely during the past 12 months  *(Yes vs. No)* |
| Suicidal ideation | During the past 12 months, did you ever seriously consider attempting suicide?  *1 “No”; 2 “Yes”* | Students who seriously considered attempting suicide in the past 12 months  *(Yes vs. No)* |
| Violence | During the past 12 months, how many times were you physically attacked?  1 *“0 time”;* 2 *“1 time” to* 8 *“12 or more time”* | Students who were physically attacked in the past 12 months  *(Yes vs. No)* |
| Self-reported experiences of bullying | During the past 30 days, on how many days were you bullied at school (or near school or on the way to and from school)?  *1 “0 day” to 7 “all 30 days”* | Students who were bullied at school in the past 30 days  *(Yes vs. No)* |
| Sexual activity | Have you ever had sexual intercourse?  0 *“No”;* 1 *“Yes”* | Students who have ever had sexual intercourse  *(Yes vs. No)* |
| Truancy | During the past 30 days, on how many days did you miss classes or school without permission?  *1 “0 day” to 5 “10 days or more”* | Students who missed any days without permission during the past 30 days  *(Yes vs. No)* |
| Alcohol consumption | During the past 30 days, on how many days did you have at least one drink containing alcohol?  1 “0 days” to 7 “All 30 days” | Students who drank at least 1 drink in the past 30 days  *(Yes vs. No)* |
| Sedentary lifestyle | How much time do you spend during a typical or usual day sitting and watching television, playing computer games, relaxing with iPad, mobile phone, talking with friends, or doing other sitting activities, such as book reading or using Facebook?  1 *“Less than 1 hour per day”;* 2 *“1 to 2 hours per day”*; 3 *“3 to 4 hours per day”* to 6 *“More than 8 hours per day”* | Student who spent three or more hours a day to do these activities  *(Yes vs. No)* |
| Low fruit/vegetable intake | - During the past 30 days, on average how many times per day did you usually eat fruit, such as a banana, apple, orange, guava, rambutan, watermelon, papaya, or mango etc.?  - During the past 30 days, on average how many times per day did you usually eat vegetables, such as morning glory, cabbage, or mustard green etc.?  *1 “I did not eat” to 7 “5 or more times per day”* | Students who ate less than 5-time fruits/vegetables per day in the past 30 days  *(Yes vs. No)* |
| People smoked in presence in the past 7 days | During the past 7 days, on how many days have people smoked in your presence (at least one time per day)?  *1 “0 days” to 5 “All 7 days”* | Students who had seen/contacted with smokers in the past 7 days  *(Yes vs. No)* |
| Smoked Shisha in the past 30 days | During the past 30 days, on how many days did you use Shisha?  *1 “0 days” to 7 “All 30 days”* | Students who smoked shisha at least 1 time in the past 30 days  *(Yes vs. No)* |

**Table 2**

*Participants’ characteristics*

| **Characteristics** | **2013** | | **2019** | |
| --- | --- | --- | --- | --- |
|  | **n** | **Weighted %** | **n** | **Weighted %** |
| **N** | **3331** |  | **7690** |  |
| **Gender** |  |  |  |  |
| Male | 1,557 | 46.9 | 3,572 | 46.0 |
| Female | 1,765 | 53.1 | 4,118 | 54.0 |
| **Age** |  |  |  |  |
| 13 | 897 | 21.7 | 1,024 | 16.7 |
| 14 | 858 | 22.5 | 1,523 | 30.4 |
| 15 | 542 | 19.6 | 1,672 | 19.9 |
| 16 | 769 | 23.9 | 1,646 | 15.9 |
| 17 | 264 | 12.3 | 1,825 | 17.1 |
| **Parental monitoring** |  |  |  |  |
| Low | 1,664 | 52.2 | 4,252 | 52.3 |
| High | 1,662 | 47.8 | 3,410 | 47.7 |
| **Parental understanding** |  |  |  |  |
| Low | 2,283 | 69.5 | 3,586 | 44.2 |
| High | 1,038 | 30.5 | 4,102 | 55.8 |
| **Parental respect** |  |  |  |  |
| Low | 1,209 | 36.8 | 1,176 | 14.3 |
| High | 2,070 | 63.2 | 6,451 | 85.7 |
| **Have close friends** |  |  |  |  |
| No | 173 | 5.5 | 778 | 8.8 |
| Yes | 3,138 | 94.5 | 6,816 | 91.2 |
| **Loneliness** |  |  |  |  |
| No | 2,910 | 88.5 | 6,589 | 87.7 |
| Yes | 367 | 11.5 | 1,098 | 12.3 |
| **Suicidal ideation** |  |  |  |  |
| No | 2,750 | 83.1 | 6,333 | 84.6 |
| Yes | 539 | 16.9 | 1,347 | 15.4 |
| **Violence** |  |  |  |  |
| No | 2,582 | 79.0 | 6,885 | 89.6 |
| Yes | 734 | 21.0 | 802 | 10.4 |
| **Bullied** |  |  |  |  |
| No | 2,456 | 77.3 | 7,258 | 94.5 |
| Yes | 744 | 22.7 | 427 | 5.5 |
| **Sexual activity** |  |  |  |  |
| No | 3,002 | 93.5 | 7,188 | 94.9 |
| Yes | 187 | 6.5 | 459 | 5.1 |
| **Truancy** |  |  |  |  |
| No | 2,725 | 80.6 | 6,307 | 84.9 |
| Yes | 598 | 19.4 | 1,191 | 15.1 |
| **Alcohol consumption in the past 30 days** |  |  |  |  |
| No | 2,476 | 75.1 | 5,781 | 77.9 |
| Yes | 722 | 24.9 | 1,864 | 22.1 |
| **Sedentary lifestyle** |  |  |  |  |
| No | 1,968 | 58.0 | 3,942 | 57.1 |
| Yes | 1,349 | 42.0 | 3,683 | 42.9 |
| **Low fruit/vegetable intake** |  |  |  |  |
| No | 693 | 20.5 | 370 | 4.6 |
| Yes | 2,603 | 79.5 | 7,295 | 95.4 |

**Table 3**

*Smoking prevalence and trend from 2013 to 2019*

| **Characteristics** | **2013** | | **2019** | |
| --- | --- | --- | --- | --- |
|  | **Weighted %** | **95% CI** | **Weighted %** | **95% CI** |
| **All** |  |  |  |  |
| Have ever smoked | 12.1 | 9.8–15.0 | 8.2 | 7.0–9.6 |
| Smoked cigarettes in the past 30 days | 4.7 | 3.5–6.3 | 2.6 | 2.0–3.4 |
| Smoked water pipe in the past 30 days | 2.4 | 1.5–3.8 | 1.0 | 0.7–1.4 |
| Traditional tobacco smoking (cigarettes and water pipe) in the past 30 days | 5.4 | 4.0–7.2 | 2.8 | 2.2–3.6 |
| People smoked in presence in the past 7 days | 75.8 | 73.4–78.0 | 66.0 | 63.8–68.2 |
| Smoked Shisha in the past 30 days | N/A | N/A | 1.3 | 0.93–1.72 |
| Smoked e–cigarettes in the past 30 days | N/A | N/A | 2.6 | 1.9–3.3 |
| **Males** |  |  |  |  |
| Have ever smoked | 19.8 | 16.8-23.3 | 13.3 | 11.0-15.9 |
| Smoked cigarettes in the past 30 days | 8.8 | 6.3-12.1 | 4.4 | 3.2-6.1 |
| Smoked water pipe in the past 30 days | 4.2 | 2.6-6.5 | 1.9 | 1.4-2.8 |
| Traditional tobacco smoking (cigarettes and water pipe) in the past 30 days | 9.6 | 6.9-13.2 | 4.9 | 3.7-6.5 |
| People smoked in presence in the past 7 days | 75.7 | 72.2-78.8 | 65.0 | 62.2-67.7 |
| Smoked Shisha in the past 30 days | N/A | N/A | 1.7 | 1.0-2.4 |
| Smoked e–cigarettes in the past 30 days | N/A | N/A | 3.6 | 2.6-4.7 |
| **Females** |  |  |  |  |
| Have ever smoked | 5.5 | 3.5-8.5 | 4.0 | 3.0-5.2 |
| Smoked cigarettes in the past 30 days | 1.1 | 0.6-2.1 | 1.0 | 0.7-1.5 |
| Smoked water pipe in the past 30 days | 0.8 | 0.4-1.9 | 0.2 | 0.1-0.5 |
| Traditional tobacco smoking (cigarettes and water pipe) in the past 30 days | 1.7 | 1.0-2.8 | 1.0 | 0.7-1.5 |
| People smoked in presence in the past 7 days | 76.1 | 73.0-78.9 | 66.9 | 64.6-69.1 |
| Smoked Shisha in the past 30 days | N/A | N/A | 1 | 0.6-1.4 |
| Smoked e–cigarettes in the past 30 days | N/A | N/A | 1.5 | 0.8-2.3 |

**Table 4**

*Factors related to traditional tobacco smoking among students aged 13–17 in Vietnam*

| **Factor** | **All students** | | **Male students** | |
| --- | --- | --- | --- | --- |
|  | **OR** | **95% CI** | **OR** | **95% CI** |
| **Year of data collection,** *(Ref: 2013)* |  |  |  |  |
| 2019 | **0.66^*^** | **0.44-0.99** | 0.63 | 0.39-1.00 |
| **Gender,** *(Ref: Female)* |  |  |  |  |
| Male | **4.25^***^** | **2.56-7.04** | **--** | **--** |
| **Age,** *(Ref: 13)* |  |  |  |  |
| 14 | 1.19 | 0.71-2.00 | 1.40 | 0.63-3.11 |
| 15 | 1.27 | 0.85-1.92 | **2.00^*^** | **1.16-3.44** |
| 16 | 1.41 | 0.86-2.30 | **2.30^**^** | **1.24-4.27** |
| 17 | 1.45 | 0.93-2.25 | **2.30^*^** | **1.18-4.48** |
| **Parental monitoring**, *(Ref: Low)* |  |  |  |  |
| High | **0.57^*^** | **0.37-0.88** | **0.58^*^** | **0.34-0.97** |
| **Parental understanding**, *(Ref: Low)* |  |  |  |  |
| High | 0.90 | 0.63-1.30 | 0.97 | 0.62-1.51 |
| **Parental respect**, *(Ref: Low)* |  |  |  |  |
| High | 0.73 | 0.51-1.04 | 0.84 | 0.57-1.24 |
| **Have close friends**, *(Ref: No)* |  |  |  |  |
| Yes | 1.28 | 0.73-2.23 | 1.19 | 0.61-2.31 |
| **Loneliness**, *(Ref: No)* |  |  |  |  |
| Yes | **1.57^*^** | **1.10-2.25** | 1.37 | 0.82-2.26 |
| **Suicidal ideation**, *(Ref: No)* |  |  |  |  |
| Yes | **1.71^*^** | **1.10-2.67** | 1.61 | 0.84-3.06 |
| **Violence**, *(Ref: No)* |  |  |  |  |
| Yes | 1.31 | 0.80-2.17 | 1.36 | 0.77-2.38 |
| **Bullied**, *(Ref: No)* |  |  |  |  |
| Yes | 0.82 | 0.50-1.33 | 0.89 | 0.50-1.59 |
| **Sexual intercourse**, *(Ref: No)* |  |  |  |  |
| Yes | **4.56^***^** | **3.17-6.57** | **4.57^***^** | **3.13-6.66** |
| **Truancy**, *(Ref: No)* |  |  |  |  |
| Yes | **2.23^**^** | **1.38-3.61** | **2.31^**^** | **1.29-4.14** |
| **Alcohol consumption in the past 30 days**, *(Ref: No)* |  |  |  |  |
| Yes | **4.17^***^** | **2.78-6.24** | **3.79^***^** | **2.31-6.22** |
| **Sedentary lifestyle**, *(Ref: No)* |  |  |  |  |
| Yes | 0.98 | 0.71-1.36 | 0.86 | 0.61-1.22 |
| **Low fruit/vegetable intake**, *(Ref: No)* |  |  |  |  |
| Yes | 1.11 | 0.73-1.67 | 1.28 | 0.71-2.31 |

^*^ *p* < 0.05; ^**^ *p* < 0.01; ^***^ *p* < 0.001

**Table 5**

*Factors related to e-cigarettes smoking among students aged 13–17 in Vietnam*

| **Factor** | **All students** | | **Male students** | |
| --- | --- | --- | --- | --- |
|  | **OR** | **95% CI** | **OR** | **95% CI** |
| **Gender,** *(Ref: Female)* |  |  |  |  |
| Male | **2.09^**^** | **1.28-3.42** |  |  |
| **Age,** *(Ref: 13)* |  |  |  |  |
| 14 | 0.56 | 0.22-1.44 | 0.57 | 0.19-1.72 |
| 15 | 0.46 | 0.17-1.25 | 0.67 | 0.21-2.13 |
| 16 | 0.42 | 0.14-1.23 | 0.56 | 0.18-1.73 |
| 17 | 0.64 | 0.21-2.00 | 1.08 | 0.33-3.50 |
| **Parental monitoring**, *(Ref: Low)* |  |  |  |  |
| High | **0.56^**^** | **0.40-0.78** | **0.54^**^** | **0.35-0.84** |
| **Parental understanding**, *(Ref: Low)* |  |  |  |  |
| High | 1.22 | 0.82-1.81 | 1.16 | 0.75-1.82 |
| **Parental respect**, *(Ref: Low)* |  |  |  |  |
| High | **0.64^*^** | **0.43-0.97** | 0.67 | 0.36-1.24 |
| **Have close friends**, *(Ref: No)* |  |  |  |  |
| Yes | 1.20 | 0.68-2.12 | 1.08 | 0.48-2.40 |
| **Loneliness**, *(Ref: No)* |  |  |  |  |
| Yes | 1.08 | 0.70-1.68 | 0.93 | 0.48-1.83 |
| **Suicidal ideation**, *(Ref: No)* |  |  |  |  |
| Yes | **1.75^*^** | **1.08-2.83** | 1.49 | 0.83-2.68 |
| **Violence**, *(Ref: No)* |  |  |  |  |
| Yes | 1.20 | 0.55-2.59 | 1.06 | 0.48-2.34 |
| **Bullied**, *(Ref: No)* |  |  |  |  |
| Yes | **2.67^**^** | **1.35-5.27** | **3.38^**^** | **1.69-6.79** |
| **Sexual intercourse**, *(Ref: No)* |  |  |  |  |
| Yes | **3.05^***^** | **1.71-5.44** | **2.97^**^** | **1.46-6.03** |
| **Truancy**, *(Ref: No)* |  |  |  |  |
| Yes | **1.68^*^** | **1.11-2.54** | 1.01 | 0.64-1.59 |
| **Alcohol consumption in the past 30 days**, *(Ref: No)* |  |  |  |  |
| Yes | **5.38^***^** | **3.75-7.71** | **3.37^***^** | **2.27-5.00** |
| **Sedentary lifestyle**, *(Ref: No)* |  |  |  |  |
| Yes | **1.81^**^** | **1.25-2.60** | **1.54^*^** | **1.05-2.27** |
| **Low fruit/vegetable intake**, *(Ref: No)* |  |  |  |  |
| Yes | 0.66 | 0.30-1.47 | 1.01 | 0.33-3.08 |

^*^ *p* < 0.05; ^**^ *p* < 0.01; ^***^ *p* < 0.001
